# Engineered Extracellular Vesicles From Human Periodontal-Ligament Stem Cells Increase VEGF/VEGFR2 Expression During Bone Regeneration

**DOI:** 10.3389/fphys.2019.00512

**Published:** 2019-04-30

**Authors:** Jacopo Pizzicannella, Agnese Gugliandolo, Tiziana Orsini, Antonella Fontana, Alessia Ventrella, Emanuela Mazzon, Placido Bramanti, Francesca Diomede, Oriana Trubiani

**Affiliations:** ^1^Department of Medical, Oral and Biotechnological Sciences, “G. d’Annunzio” University of Chieti–Pescara, Chieti, Italy; ^2^IRCCS Centro Neurolesi “Bonino Pulejo”, Messina, Italy; ^3^Institute of Cell Biology and Neurobiology, National Research Council, Rome, Italy; ^4^Department of Pharmacy, “G. d’Annunzio” University of Chieti–Pescara, Chieti, Italy

**Keywords:** mesenchymal stem cells, bone regeneration, VEGF, VEGFR2, collagen membrane, extracellular vesicles, polyethylenimine

## Abstract

Bone regeneration represents still a challenge, in particular for calvarium defects. Recently, the development of biomaterials with the addiction of stem cells is giving promising results for the treatment of bone defects. In particular, it was demonstrated that scaffolds enriched with mesenchymal stem cells (MSCs) and/or their derivatives, such as conditioned medium (CM) and extracellular vesicles (EVs), may improve bone regeneration. Moreover, given the deep link between osteogenesis and angiogenesis, a successful approach must also take into consideration the development of vascularization. In this work we evaluated the bone regeneration capacity of a collagen membrane (3D-COL) enriched with human periodontal-ligament stem cells (hPDLSCs) and CM or EVs or EVs engineered with polyethylenimine (PEI-EVs) in rats subjected to a calvarial defect. We evaluated also their capacity to induce angiogenic factors. At first, *in vitro* results showed an increased expression of osteogenic markers in hPDLSCs cultured with the 3D-COL and PEI-EVs, associated also with the increased protein levels of Vascular endothelial growth factor (VEGF) and VEGF receptor 2 (VEGFR2). The increased expression of these proteins was confirmed also *in vivo* in rats implanted with the 3D-COL enriched with hPDLSCs and PEI-EVs. Moreover, histological examination evidenced in this group of rats the activation of bone regeneration and of the vascularization process. Also MicroCT imaging with morphometric analysis confirmed in rats transplanted with 3D-COL enriched with hPDLSCs and PEI-EVs an important regenerative process and a better integration level. All together, these results evidenced that the 3D-COL enriched with hPDLSCs and PEI-EVs may promote bone regeneration of calvaria defects, associated also with an increased vascularization.

## Introduction

Bone defects, that may be caused by trauma, malformations, tumor resection, have a great negative impact on the patients’ quality of life and bone regenerative medicine could be a promising approach for these patients (Dimitriou et al., 2011). Indeed, even if bone tissue has a great capacity to repair, in some conditions bone regeneration is needed in large quantity. Moreover, also the age may be important, for example patients less than 2 years old may regenerate calvarial defects, on the contrary, elderly patients possess minor regenerative capacity (Szpalski et al., 2010). Autologous bone grafts represent a gold-standard but show many limitations and for this reason the development of new approaches based on the use of scaffolds and/ or stem cells is under research (Dimitriou et al., 2011; Jin and Lee, 2018; White and Olabisi, 2019). In particular, mesenchymal stem cells (MSCs) and their derivatives, such as conditioned medium (CM) and extracellular vesicles (EVs), in association with biomaterials have shown to be able to regenerate bone tissues, including calvaria defects (Diomede et al., 2018a,b,c,d).

Bone regeneration is a process of great complexity, that is based on the interaction between various cell types. In particular, bones are highly vascularized tissues and it is clear that osteogenesis and angiogenesis are two processes deeply linked. The most exciting challenge in regenerative medicine is to find a scaffold that ensure the regenerative healing bone (Tatullo et al., 2019). Blood vessels play a role as transporters of growth factors, minerals and others into the osteogenic microenvironment. However, they are also needed as a structural template around which bone generation starts (Grosso et al., 2017).

Furthermore, blood vessels exerted another function known as angiocrine function, that through paracrine signaling modulate growth, differentiation and regeneration of several cells, such as bone, then promoting osteogenesis (Rafii et al., 2016; Ramasamy et al., 2016). Vascularization represents an aspect that must be taken into consideration in the field of bone regenerative medicine, that try to develop bone substitutes in order to replace bone loss caused by pathological conditions or trauma, when the physiological bone regeneration is not enough. Indeed, after the implantation of the bone graft *in vivo*, in order to maintain cell viability it is necessary the development of blood vessels because poor blood perfusion results in oxygen and nutrient deficiency and as a consequence to cell death (Grosso et al., 2017). Blood vessels and bone communicate both during physiological development and fracture healing or bone regeneration (Clarkin and Gerstenfeld, 2013). Furthermore, osteoblasts are able to produce pro-angiogenic factors, including vascular endothelial growth factor (VEGF) (Hu and Olsen, 2016).

Vascular endothelial growth factor A plays a main role in angiogenesis, but it is important also for bone growth and regeneration, coupling the 2 processes. Indeed, VEGF on one hand induces the migration and proliferation of endothelial cells and on the other hand it stimulates osteogenesis regulating osteogenic growth factors (Grosso et al., 2017).

The VEGF family is composed of different members, but VEGF-A, commonly named VEGF, was the first member to be discovered and have a main role in angiogenesis. Other than from endothelial cells VEGF is released also from other cell types, including osteoblasts (Melincovici et al., 2018). VEGF binds to the extracellular domains of two tyrosine kinase receptors, VEGF receptor 1 (VEGFR1) and VEGF receptor 2 (VEGFR2) (Karaman et al., 2018). VEGF-A signal in blood endothelial cells seems to be mainly mediated through the activation of VEGFR2 (Simons et al., 2016). VEGFR2 plays a role in angiogenesis, proliferation, differentiation and migration of endothelial cells (Melincovici et al., 2018).

Given the important role played by angiogenesis in the bone, an approach able to increase bone regeneration together with vascularization may be the optimal treatment for the repair of bone defects.

In this work, we evaluated the regeneration of calvaria in rats transplanted with a collagen membrane (3D-COL) enriched with MSCs, namely human periodontal ligament stem cells (hPDLSCs), and/or their derivatives, such as CM and EVs. We tested also engineered EVs, that were coated with polyethylenimine (PEI), in order to evaluate if their performance improved compared to normal EVs.

PEI is a polymer that present in its structure protonatable amino groups (Boussif et al., 1995). Thanks to the highly dense positive charge it is widely used to deliver DNA and RNA both *in vitro* and *in vivo*, showing the ability to form non-covalent complexes with DNA promoting its delivery (Werth et al., 2006). This action is probably due to PEI capacity to induce the intracellular release of the complexes with nucleic acids from endosomes, thanks to the induction of osmotic swelling (i.e., proton-sponge effect) that causes the burst of endosomes without the need for an additional endosomolytic agent (Zuber et al., 2001). Thus, the engineering of EVs (PEI-EVs) with PEI, should promote the release of EV content into cells.

In particular, our aim was to develop a new construct able to promote bone regeneration but also the expression of pro-angiogenic factors with consequent vascularization, given the important role played by angiogenesis for bone growth and regeneration.

## Materials and Methods

### Scaffold Material

We employed the 3D-COL made of consistency dense collagen fiber derived from equine mesenchymal tissue (Evolution; Tecnoss^®^ Dental, Giaveno, Italy). It is adapt for both hard and soft tissue, and it allows easy and secure suturability of nearby tissue, and sufficient protection of underlying grafts. Sterile scalpels were used in order to cut pieces of about 4 x 7 mm. 3D-COL pieces were washed with sterile phosphate buffered saline (PBS) (Lonza, Basel, Switzerland) to permit rehydration of the material before use.

### *In vitro* Analysis

#### Ethics Statement for *in vitro* Experiments

The study was performed in accordance with the guidelines of the Helsinki Declaration (2013). The written approval for the human periodontal ligament collection performed in this study has been obtained from the Medical Ethics Committee at the Medical School, “G. d’Annunzio” University of Chieti–Pescara, Chieti, Italy (n°266/17.04.14, Principal Investigator: Trubiani Oriana). The written informed consent, for clinical research and for the processing of personal data, was obtained from all subjects before sample collection. The Department of Medical, Oral and Biotechnological Sciences and the Laboratory of Stem Cells and Regenerative Medicine are certified according to the quality standard ISO 9001:2008 RINA (certificate no. 32031/15/S).

#### Cell Culture Establishment

Five different persons in healthy general conditions were selected to remove the teeth for orthodontic purpose. After, the cells were cultured using the chemically defined MSCGM-CD^TM^ BulletKit media (MSCGM-CD), (Lonza), that was changed twice a week, in order to permit the growth of human MSCs and to minimize the exposure to non-human substances. Cells were isolated and characterized as previously described (Diomede et al., 2016a, 2017; Rajan et al., 2016). Briefly cells were isolated from periodontal ligament, small fragments were placed in Petri dish and cultured with MSCGM-CD in a humidified atmosphere with 5% CO_2_ at 37°C. After 2 weeks cells spontaneously migrated to the bottom dish. Cells at second passage were used for the following experiments. Cell morphology was observed under light microscopy Leica DMIL (Leica Microsystem, Milan, Italy).

#### Cytoflorimetric Study

Antibodies. Fluorescein isothiocyanate-conjugated anti-CD13 (CD13 FITC), phycoerythrin-conjugated anti-CD29 (CD29 PE), FITC-conjugated: anti-CD45 (CD45-FITC), anti-CD105 (CD105 FITC) were obtained from Ancell; FITC-conjugated anti-CD14 (CD14 FITC) was purchased from Milteny Biotec; PE-conjugated anti-CD73 (CD73 PE), FITC-conjugated anti-CD90 (CD90 FITC), Alexa488-conjugated anti-Sox2 (Sox2 Alexa488), FITC-conjugated anti-SSEA-4 (SSEA-4 FITC) and PE-conjugated anti-OCT3/4 (OCT3/4 PE) were obtained from Becton Dickinson; PE-conjugated anti-CD34 (CD34-PE) was purchased from Beckman Coulter; and an appropriate secondary FITC-conjugated antibody was obtained from Jackson Immunoresearch Laboratories. Washing buffer (phosphate-buffered saline, PBS, 0.1% sodium azide, and 0.5% bovine serum albumine, BSA) was used for all washing steps (3 mL of washing buffer and centrifugation, 400 g 8 min at 4°C). Briefly, 5 × 10^5^ cells/sample were incubated with 20 mM ethylenediaminetetraacetic acid (EDTA) at 37°C for 10 min and washed. Staining of surface antigens and intracellular antigens was carried out according to Diomede et al. (2018d). Quality control included a regular check-up with Rainbow Calibration Particles (BD Biosciences). Debris was excluded from the analysis by gating on morphological parameters; 20,000 non-debris events in the morphological gate were recorded for each sample. To assess non-specific fluorescence, we used specific irrelevant controls. All antibodies were titrated under assay conditions, and optimal photomultiplier (PMT) gains were established for each channel. Data were analyzed using FlowJo software (TreeStar) (Trubiani et al., 2013). mean fluorescence intensity ratio (MFI Ratio) was calculated by dividing the MFI of positive events by the MFI of negative events.

#### Cell Proliferation and Viability Assay

hPDLSCs at passage 2 were seeded at 1 × 10^3^ cells/well in triplicate using a 96-well flat-bottom plate and maintained in culture medium for 24, 48, 72 h and 1 week. After the incubation period, 15 μL/well of MTT solution were added to culture medium and cells were incubated for 3 h at 37°C. The supernatants were read at 650 nm wavelength using a microplate reader (Synergy HT; BioTek Instruments). Moreover, the doubling time of the trypan blue harvested cells, at 24, 48, 72 h and 1 week of culture, was calculated by using an algorithm available online^1^.

#### Conditioned Medium (CM) Collection

After 48 h, the CM was collected from 15 × 10^3^/cm^2^ hPDLSCs at passage 2. The CM was centrifuged at 1200 rpm for 5 min in order to eliminate suspension cells and debris. The supernatants were centrifuged at 3000 rpm for 3 min, followed by collection of the secondary supernatants. After, 1 mL of secondary supernatants was resuspended in 3 mL of ice aceton and maintained over night at 4°C, and after centrifuged at 16,000 rpm for 12 min at 4°C (Centrifuge 5804 R, Eppendorf, Milan, Italy) (Giacoppo et al., 2017). The suspension was lysated in RIPA and quantified by means Bradford assay. Total proteins obtained were 125 μg/μL.

#### hPDLSCs Extracellular Vesicles (EVs) Isolation

After 48 h, the CM was collected from 15 × 10^3^/cm^2^ hPDLSCs at 2nd passage and centrifuged at 3000 × g for 15 min to remove suspension cells and debris. For the EVs extraction, an exoquick TC commercial agglutinant (System Biosciences, Palo Alto, CA, United States) was used. Briefly, 2 ml of ExoQuick TC were added to 10 ml of CM collected from hPDLSCs. The mix was incubated overnight at 4°C without rotation. A centrifugation was performed at 1,500× *g* for 30 min to sediment the EVs. The pellets were resuspended in 200 μl of PBS. The EVs, splitted in two aliquots, were precipitated and quantification of whole homogenate proteins was used as a confirmation of the presence of the release of EVs in hPDLSCs.

#### Engineered EVs Preparation

The EVs were engineered by coating EVs with branched polyethylenimine (PEI, MW 25,000 Sigma-Aldrich) by using a non-covalent layer-by-layer protocol (Angelini et al., 2007, 2008). The EVs pellet (100 μL), dispersed with 2 mL of PBS, was added of 2 mL of PEI dissolved in 0.3 M NaCl (final concentration of PEI 0.05 mg/mL) and incubated for 20 min at room temperature. The concentration was chosen because demonstrated to be the best compromise between activity and toxicity (Diomede et al., 2018a).

The obtained suspension was centrifuged at 4000 rpm for 15 min, the supernatant was removed in order to get rid of excess PEI and the precipitate was resuspended in 2 mL of PBS. The EVs suspension was characterized by using dynamic light scattering (DLS) experiments (Brookhaven Instruments Corporation, 90Plus, Holtsville, New York, United States).

To evaluate the interaction between EVs or PEI-EVs and hPDLSCs, WGA Alexa Fluor 488 stained EVs and PEI-EVs were analyzed at confocal laser scanning microscope (CLSM) (LSM800; Zeiss, Jena, Germany) level after 24 h of incubation.

#### Atomic Force Microscope (AFM) Measurements

In order to analyze the morphology of EVs and PEI-EVs, atomic force microscopy (AFM) analyses were performed by using the Multimode 8 AFM microscope with Nanoscope V controller (Bruker, Palaiseau, France). A silicon cantilever and a RTESPA-150 tip (cantilever resonance frequency 150 kHz and spring constant 5 N/m) with a tip radius of 8 nm were used in a ScanAsyst^TM^ in air mode. A scan size area of 5 μm × 5 μm was performed. The samples were prepared by depositing a drop of solution of EVs or PEI-EVs on SiO2 wafer followed by drying in the oven at 37°C for 2 h and then at room temperature overnight (Diomede et al., 2018c).

#### RNA Isolation and Real-Time-PCR Analysis

hPDLSCs were seeded at 80 × 10^3^/well in 6-well plates in basal medium alone or in the presence of 3D-COL, 3D-COL/CM, 3D-COL/EVs or 3D-COL/PEI-EVs. Cells in each condition were maintained in culture for 28 days and, after harvesting, total RNA was isolated using the Total RNA Purification Kit (Norgen Biotek Corp., Ontario, CA, United States) according to the manufacturer’s instructions. The M-MLV Reverse Transcriptase reagents (Applied Biosystems) were used to generate cDNA. Real-Time PCR was carried out with the Mastercycler ep realplex real-time PCR system (Eppendorf, Hamburg, Germany). hPDLSCs expression of Runt-related transcription factor-2 (RUNX-2) Collagen 1A1 (COL1A1), Bone Morphogenetic Protein 2/4 (BMP2/4), VEGFA and Vascular Endothelial Growth Factor Receptor 2 (VEGFR2) was evaluated after 28 days in basal medium. Gene Expression assay was performed as previously described (Diomede et al., 2018e). RT-PCR was performed in three independent experiments, duplicate determinations were carried out for each sample.

#### Western Blot Analysis

hPDLSCs were seeded at 80 × 10^3^/well in 6-well plates in basal medium alone or in the presence of 3D-COL, 3D-COL/CM, 3D-COL/EVs or 3D-COL/PEI-EVs. After 28 days in culture, cells were harvested and proteins were collected from 3D-COL/hPDLSCs, 3D-COL/hPDLSCs/CM, 3D-COL/hPDLSCs/EVs and 3D-COL/hPDLSCs/PEI-EVs samples (40 μg/sample). The western blot procedure was performed as previously reported (Ballerini et al., 2017). VEGFA (Santa Cruz Biotechnology; 1:1000) and VEGFR2 (Santa Cruz Biotechnology; 1:1000) were used as primary antibody. β-Actin (Santa Cruz Biotechnology; 1:750) was used to assess the uniform protein loading (Libro et al., 2016). Bands were analyzed by the ECL method using Alliance 2.7 (UVItec Limited, Cambridge, United Kingdom). Protein bands were quantified with a computer program (ImageJ software). Entire blots were visible in Supplementary Figure S1.

### *In vivo* Analysis

#### Animals

Male Wistar rats weighing 300–350 g were used for this experiment. Animals were acquired from Harlan Milan, Italy and housed in individually ventilated cages and maintained under 12 h light/dark cycles, at 21 ± 1°C and 50–55% humidity with food and water *ad libitum*.

#### Ethics Statement for Animal Use

All animal care and use was accomplished according to the European Organization Guidelines for Animal Welfare. The study has been authorized by the Ministry of Health “General Direction of animal health and veterinary drug” (Authorization 768/2016-PR 28/07/2016- D.lgs 26/2014). The experiments were planned in such a way to minimize the total number of rats needed for the study. We used G^∗^Power software for power analysis, applying *F*-test one-way ANOVA for *a priori* analysis. Considering an effect size = 1, α error = 0.05, power (1- β error) = 0.80 and five groups, we obtained a total sample size of 20 (*n* = 4 for each group) (Supplementary Figure S2).

#### The Implant of Scaffold

To implant the scaffold, rats were anesthetized with a mix of tiletamine and xylazine (10 ml/kg, intraperitoneal; i.p.) and the implant site was prepared with iodopovinone (Betadine). Following trichotomy, a median sagittal incision of about 1.0 cm in the frontoparietal region, a total thickness cut was applied; the calvaria was then exposed and the rectangular section bone receiving site, with a diameter of 4 mm and a height of 0.25 mm, was injured by means of a dedicated rotary instrument at a controlled speed (trephine milling machine, Alpha Bio-Tec, Siena, Italy) under constant irrigation of a physiological solution.

Thanks to their texture and flexibility, the following: 3D-COL, 3D-COL/hPDLSCs, 3D-COL/hPDLSCs/CM, 3D-COL/hPDLSCs/EVs and 3D-COL/hPDLSCs/PEI-EVs were easily inserted into contact with bone tissue to cover the damaged area. The skin flap was then sutured with small absorbable sutures of reduced diameter (Caprosyn 6-0), using interrupted points. Standard feeding and hydration were maintained constant throughout the post-operative phase.

#### Experimental Design

Rats were randomly distributed into the following groups:

(1) 3D-COL (*N* = 4): rats subjected to the scraping of the cortical calvaria bone tissue and implant of 3D-COL;
(2) 3D-COL/hPDLSCs (*N* = 4): rats subjected to scraping of the cortical calvaria bone tissue and implant of 3D-COL enriched with hPDLSCs;
(3) 3D-COL/hPDLSCs/CM (*N* = 4): rats subjected to scraping of the cortical calvaria bone tissue and implant of 3D-COL enriched with hPDLSCs and CM;
(4) 3D-COL/hPDLSCs/EVs (*N* = 4): rats subjected to scraping of the cortical calvaria bone tissue and implant of 3D-COL enriched with hPDLSCs and EVs;
(5) 3D-COL/hPDLSCs/PEI-EVs (*N* = 4): rats subjected to the scraping of the cortical calvaria bone tissue and implant of 3D-COL enriched with hPDLSCs and PEI-EVs;

Animals received as anti-inflammatory/analgesic treatment Carprofen (5 mg/Kg, subcutaneous).

After 6 weeks the animals were euthanized, after anesthetic premedication (tiletamine and zolazepam), by intravenous administration of Tanax (5 ml/kg body weight) and their calvariae were processed for morphological analysis.

#### Histological Evaluation

The samples were fixed for 72 h in 10% formalin solution, dehydrated in ascending graded alcohols and embedded in LR White resin (Sigma-Aldrich) (Pizzicannella et al., 2011). Following polymerization, undecalcified oriented cut sections of 50 μm were obtained and ground down to about 30 μm by using the TT System (TMA2, Grottammare, Italy). The sections were analyzed with the CLSM LSM510 META (Zeiss) and, after staining with a solution of acid fuchsine and methylene blue, they were observed at light microscopy. The investigation was carried out by means of a bright-field light microscope (Leica Microsystem, Milan, Italy) connected to a high-resolution digital camera DFC425B Leica (Leica Microsystem).

#### Immunofluorescence Analysis

Semithin non-decalcified sections, embedded in LR white resin, of all samples were prepared for immunofluorescence analysis. Specimens were blocked with 3% of BSA in PBS-tween for 1 h. Primary monoclonal antibody anti-human VEGF (1:100, mouse) and VEGFR2 (1:100, mouse) were used, followed by Alexa Fluor 488 green fluorescence conjugated goat anti-mouse (Diomede et al., 2018a). The sections were analyzed with the CLSM LSM800 Axiovert (Zeiss, Jena, Germany).

#### MicroCT Evaluation

Tomographic analysis were performed using a high-resolution 3D Micro-CT Imaging System (Skyscan 1172G Bruker, Kontich, Belgium), characterized by a L7901-20 Microfocus X-ray Source (Hamamatsu Photonics Italia srl, Rome, Italy). Computed tomography images were acquired with 0.5 mm Al filter, image pixel/size of 7.4 um, camera binning 2×2, tube voltage peak of 49 kV, tube current of 200 uA, exposure time of 820 ms. Reconstructions of the acquired 2D images, about 1300 slices per sample, in volume images were performed using built-in NRecon Skyscan reconstruction software (Version: 1.6.6.0; Skyscan Bruker, Billerica, MA, United States). The reconstructed tomographic 3D datasets were generated using 3D Visualization Softwares CTvox v.2.5 and DataViewer v.1.4.4 (Skyscan Bruker, Billerica, MA, United States) to the volume rendering and virtual sectioning views. Using Bruker CT-Analyser software Version 1.13 (CTAn), a volume of interest (VOI) of 300 slides has been extrapolated from each dataset, corresponding to the central zone and identical for each sample, starting from a ROI (Region of Interest) of 6 × 4 mm^2^ that included the damage, for automated 3D measurements of bone parameters. The bone volume (BV) inside the original defect site were quantitatively analyzed by μCT as previously described (Lin et al., 2014). Also percent BV (BV/tissue volume, TV), bone surface (BS in 2D), bone specific surface (BS/BV), bone surface density (BS/TV), connectivity and Euler number were evaluated in the same area. Data were expressed as the mean ± SD values.

### Data and Statistical Analysis

Data were expressed as means and standard deviation of the recorded values. Statistical analysis was performed using GraphPad Prism version 7.0 software (GraphPad Software, La Jolla, CA, United States). The Shapiro-Wilk normality test could not be performed with *n* = 3 (for *in vitro* experiments) and *n* = 4 replicates (for *in vivo* experiments), for this reason we applied non-parametric test. In particular, Kruskal-Wallis test followed by Dunn’s multiple comparison test was performed. Differences were considered significant when *p* < 0.05.

## Results

### hPDLSCs Characterization

**Figure 1:**
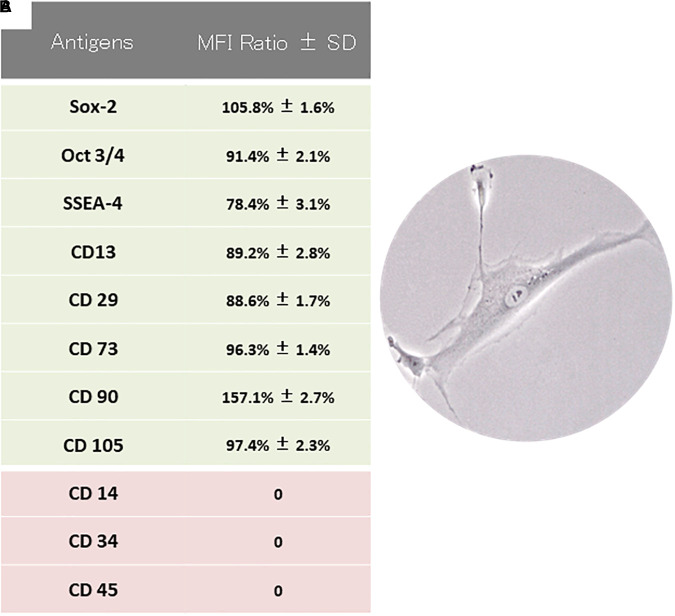
hPDLSCs characterization. **(A)** Cytofluorimetric analysis of hPDLSCs culture. **(B)** Light microscopy representative picture of hPDLSCs adherent to the culture dish.

hPDLSCs were characterized by means flow cytometry analysis. Cells were negative for any hematopoietic marker (CD14, CD34, and CD45). On the contrary, they expressed a variety of mesenchymal markers (CD13, CD29, CD73, CD90, and CD105) and the stemness markers as Sox-2, Oct 3/4 and SSEA-4 (Figure 1A). Cells observed under light microscopy showed a fibroblastic like morphological shape (Figure 1B). MTT assay was performed at 24, 48, 72 h and 1 week. The obtained data displayed an increasing trend in the proliferation rate for all the examined time points (24 h: 1.35 ± 0.26; 48 h: 1.85 ± 0.2; 72 h: 2.67 ± 0.29; 1 week: 4.29 ± 0.36). Cell growth showed the logarithmic rate. The previous results were also confirmed through the analysis of Trypan Blue exclusion test (24 h: 4325 ± 354; 48 h: 8524 ± 652; 72 h: 12487 ± 859; 1 week: 23568 ± 1201).

### EVs and PEI-EVs (AFM)

**Figure 2:**
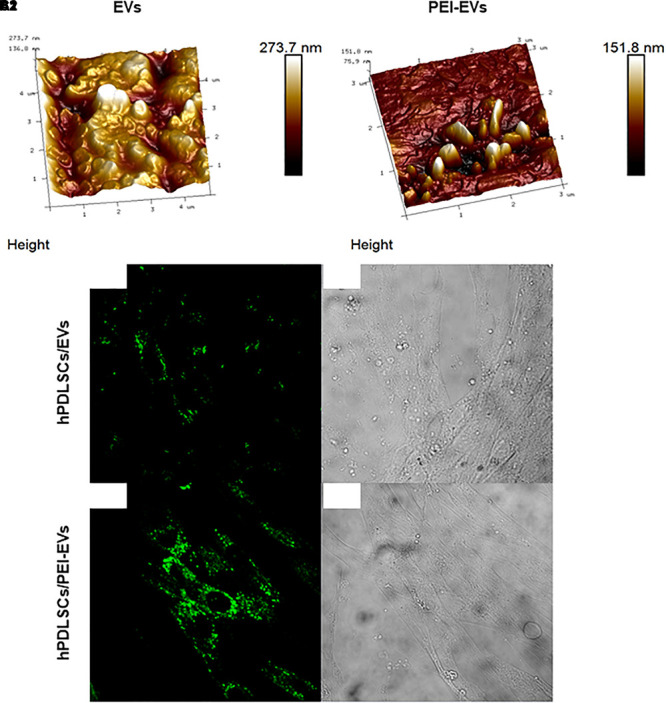
EVs and PEI-EV atomic force microscopic evaluation. **(A1)** EVs analyzed by tapping mode topographic 3D AFM technique showed a globular shape with a central depression. **(A2)** PEI-EVs analyzed by tapping mode topographic 3D AFM technique, showed a globular morphology with a less smooth surface. **(B1)** hPDLSCs incubated with WGA Alexa Fluor 488 stained EVs (green). **(C1)** hPDLSCs incubated with WGA Alexa Fluor 488 stained PEI-EVs (green). **(B2**,**C2)** Cell morphology observed at light transmission channel (gray scale). Mag, 63X.

The DLS analysis showed the presence of a heterogeneous population of EVs, spanning from 100 to 710 nm for the pure EVs and from 1050 to 7700 nm for the engineered PEI-EVs, as already previously highlighted (Diomede et al., 2018a). The observed dimensional increase evidenced the occurred coating. It is worth noting that these measurements are strongly affected by the biggest vesicles that hide the smaller ones. For this reason, EVs and PEI-EVs were also analyzed by AFM. Figure 2A1 highlights the presence of a large number of globular EVs of different dimensions. Among them, a few EVs present a characteristic central depression, thus confirming previous reports on the shape of EVs (Sharma et al., 2010). EVs appear mainly aggregated due to the fact that the preparation of the sample required solvent removal and this aggregation does not allow to estimate, through this technique, the real size of each EV. Figure 2A2 evidences engineered PEI-EVs characterized by an elongated spherical morphology with no central depression and a less smooth surface with respect to bare EVs, likely due to a more rigid structure consequent to PEI coating.

### hPDLSCs and EVs/PEI-EVs Interaction

The EVs and PEI-EVs were stained with WGA Alexa Fluor 488 and incubated with hPDLSCs for 24 h. The CLSM observations were carried out after 24 h of culture (Figure 2B1,C1,B2,C2, Supplemetary Figure S3). CLSM images showed the presence of EVs and PEI-EVs at cytoplasmic level. In particular, in the cytoplasm of hPDLSCs, PEI-EVs showed a higher concentration compared to the EVs.

### Gene Expression of Osteogenic Markers and VEGF and VEGFR2 *in vitro*

**Figure 3:**
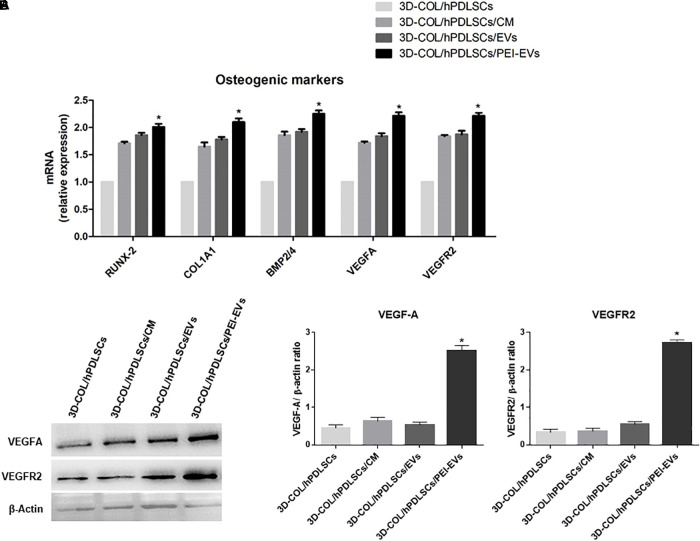
Gene expression of osteogenic markers and gene and protein expression for VEGF and VEGFR2 *in vitro* (*n* = 3). **(A)** Bar charts showed the gene expression for RUNX-2, COL1A1, BMP2/4, VEGF, and VEGFA in 3D-COL/hPDLSCs, 3D-COL/hPDLSCs/CM, 3D-COL/hPDLSCs/EVs, 3D-COL/hPDLSCs/PEI-EVs. Results showed the significant upregulation of these markers in 3D-COL/hPDLSCs/PEI-EVs compared to 3D-COL/hPDLSCs. **(B)** Protein specific bands and densitometric analysis of VEGFA and VEGFR2 in 3D-COL/hPDLSCs, 3D-COL/hPDLSCs/CM, 3D-COL/hPDLSCs/EVs, 3D-COL/hPDLSCs/PEI-EVs. Western blot analysis of VEGF and VEGFR2 *in vitro* evidenced increased levels for both proteins in 3D-COL/hPDLSCs/PEI-EVs compared to the other groups. ^∗^*p* < 0.05 3D-COL/hPDLSCs/PEI-EVs compared to 3D-COL/hPDLSCs.

In order to evaluate if the addition of CM, EVs or PEI-EVs influenced the differentiation capacity of hPDLSCs cultured on the 3D-COL in basal culture *in vitro*, we evaluated the gene expression of osteogenic markers through real-time PCR. We evaluated also their capacity to induce the expression of VEGF and VEGFR2. We observed that the gene expression of RUNX2, COL1A1, BMP2/4, VEGFA and VEGFR2 was up regulated in 3D-COL/hPDLSCs/EVs, 3D-COL/hPDLSCs/PEI-EVs and 3D-COL/hPDLSCs/CM when compared to 3D-COL/hPDLSCs (Figure 3A). However, the difference was statistically significant only for 3D-COL/hPDLSCs/PEI-EVs compared to 3D-COL/hPDLSCs. Indeed, the gene expression for these markers was about 2-fold higher in 3D-COL/hPDLSCs/PEI-EVs compared with 3D-COL/hPDLSCs.

### Western Blot Analysis of VEGF and VEGFR2 Levels *in vitro*

Given the important role played by VEGF in bone regeneration, we evaluated the capacity of hPDLSCs and their derivatives to increase the protein levels of VEGF and its receptor VEGFR2 *in vitro*. Western blot results showed a significant up regulation of VEGF and VEGFR2 in 3D-COL/hPDLSCs/PEI-EVs compared to 3D-COL/hPDLSCs (Figure 3B). Beta actin has been used as internal control.

### Immunofluorescence for VEGFA and VEGFR2 *in vivo*

**Figure 4:**
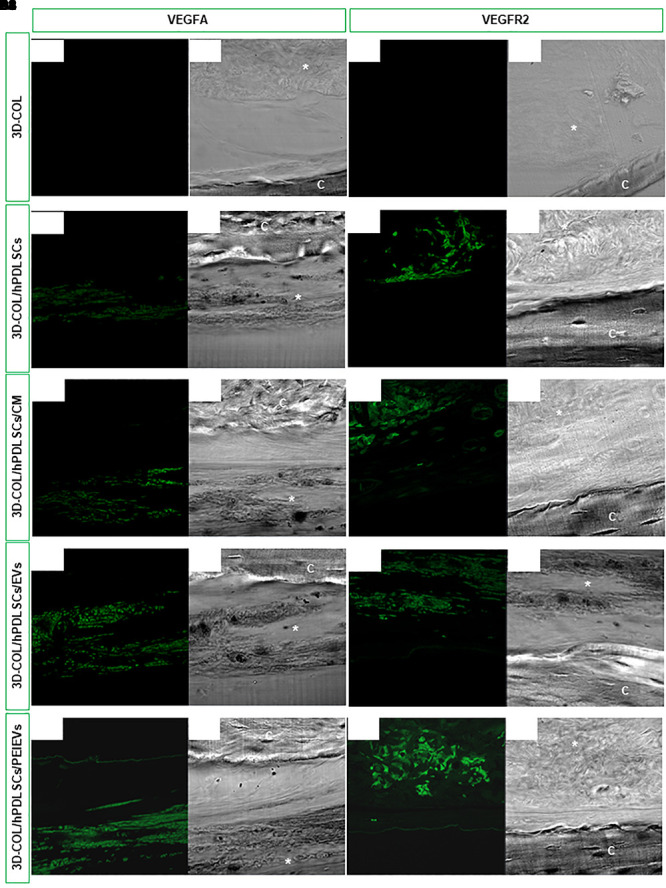
*In vivo* VEGFA and VEGFR2 expression. Immunofluorescence staining of VEGFA and VEGFR2 showed the presence of the protein in semithin section samples obtained after 6 weeks of grafting in rat calvaria. Panels (**A1,A2**, **B1,B2**, **C1,C2**, **D1,D2**, **E1,E2)** showed the expression of VEGFA. Panels (**A3,A4**, **B3,B4**, **C3,C4**, **D3,D4**, **E3,E4)** showed the expression of VEGFR2. **(A1,A3)** 3D-COL at green fluorescent channel, **(A2,A4)** 3D-COL at light transmission channel. **(B1,B3)** 3D-COL/hPDLSCs at green fluorescent channel, **(B2,B4)** 3D-COL /hPDLSCs at light transmission channel. **(C1,C3)** 3D-COL/hPDLSCs/CM at green fluorescent channel, **(C2,C4)** 3D-COL/hPDLSCs/CM at light transmission channel. **(D1,D3)** 3D-COL/hPDLSCs/EVs at green fluorescent channel, **(D2,D4)** 3D-COL/hPDLSCs/EVs at light transmission channel. **(E1,E3)** 3D-COL/hPDLSCs/PEI-EVs at green fluorescent channel, **(E2,E4)** 3D-COL/hPDLSCs/PEI-EVs at light transmission channel. Mag, 20X; C, mouse calvaria; ^∗^3D-COL.

The expression of VEGFA and VEGFR2 *in vivo* was evaluated on calvaria sections of 3D-COL, 3D-COL/hPDLSCs, 3D-COL/hPDLSCs/CM, 3D-COL/hPDLSCs/EVs and 3D-COL/hPDLSCs/PEI-EVs groups using CLSM. VEGF and VEGFR2 were expressed in 3D-COL/hPDLSCs, 3D-COL/hPDLSCs/CM, 3D-COL/hPDLSCs/EVs and 3D-COL/hPDLSCs/PEI-EVs (Figure 4). In 3D-COL grafted samples the fluorescence was obviously negligible (Figure 4A1,A3). VEGFA and VEGFR2 were over-expressed in 3D-COL/hPDLSCs/PEI-EVs when compared to 3D-COL/hPDLSCs, 3D-COL/hPDLSCs/CM and 3D-COL/hPDLSCs/EVs (Figure 4). This results validated a possible correspondence *in vitro* and *in vivo* conditions.

### Histological Evaluation

**Figure 5:**
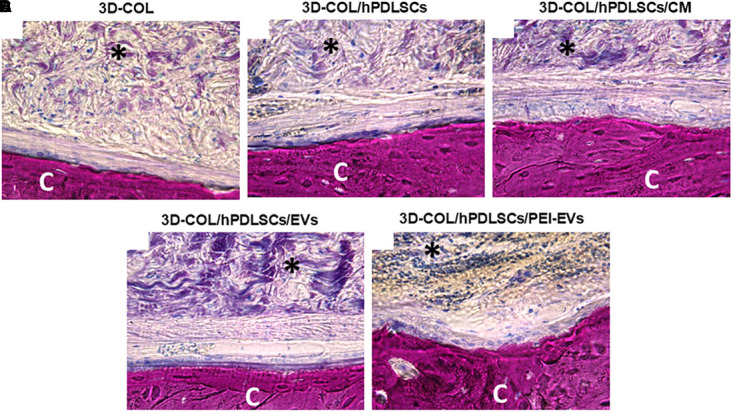
Histologic evaluation. Histologic view observed at light microscopy after 6 weeks of grafting and stained with acid fuchsin and methylene blue solution. **(A)** 3D-COL. **(B)** 3D-COL/hPDLSCs. **(C)** 3D-COL/hPDLSCs/CM. **(D)** 3D-COL/hPDLSCs/EVs. **(E)** 3D-COL/hPDLSCs/PEI-EVs. Mag, 40X; C, mouse calvaria; ^∗^3D-COL.

Histological evaluations were carried out after 6 weeks of grafting showing a better regeneration capacity of 3D-COL/hPDLSCs/PEI-EVs (Figure 5).

The evaluation of all samples compared to 3D-COL transplantation group (Figure 5A) showed a better integration of the membrane in the defect site. In particular, 3D-COL/hPDLSCs showed a well integration with the presence of abundant extracellular matrix (Figure 5B). The same results were obtained with 3D-COL/hPDLSCs/CM, on the surface of the native bone osteoblast like cells were visible (Figure 5C).

In 3D-COL/hPDLSCs/EVs and 3D-COL/hPDLSCs/PEI-EVs the vascularization process was evident other than an organized extracellular matrix with no inflammatory reaction. In particular, 3D-COL/hPDLSCs/PEI-EVs group showed the better performance in terms of integration and regenerative capacity. Osteoclasts and osteoblasts were visible on the native bone indicating an active bone regeneration process (Figure 5E).

### MicroCT

**Figure 6:**
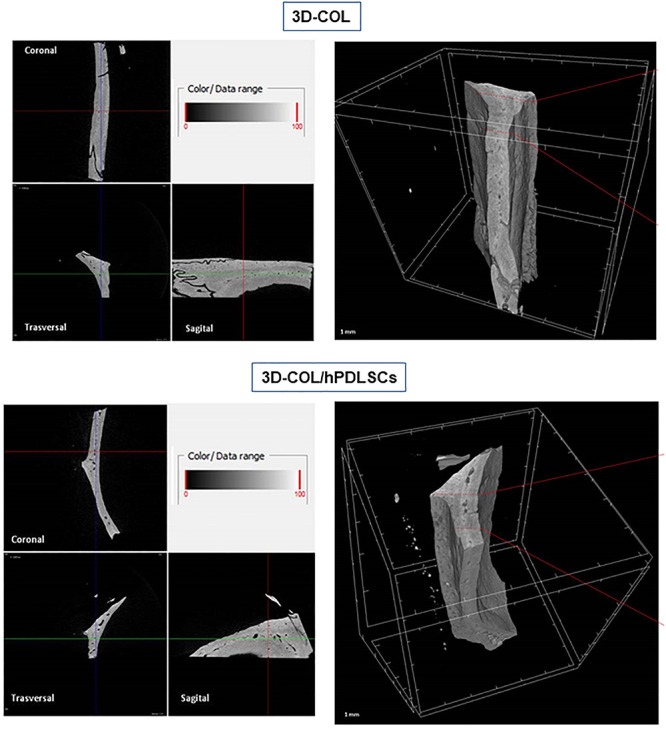
Micro-computed tomography (CT) analyses. 3D virtual histologic evaluation in volume rendering visualization modality of 3D-COL and 3D-COL/hPDLSCs, with 2D virtual sectioning in the 3 orthogonal planes.

**Figure 7:**
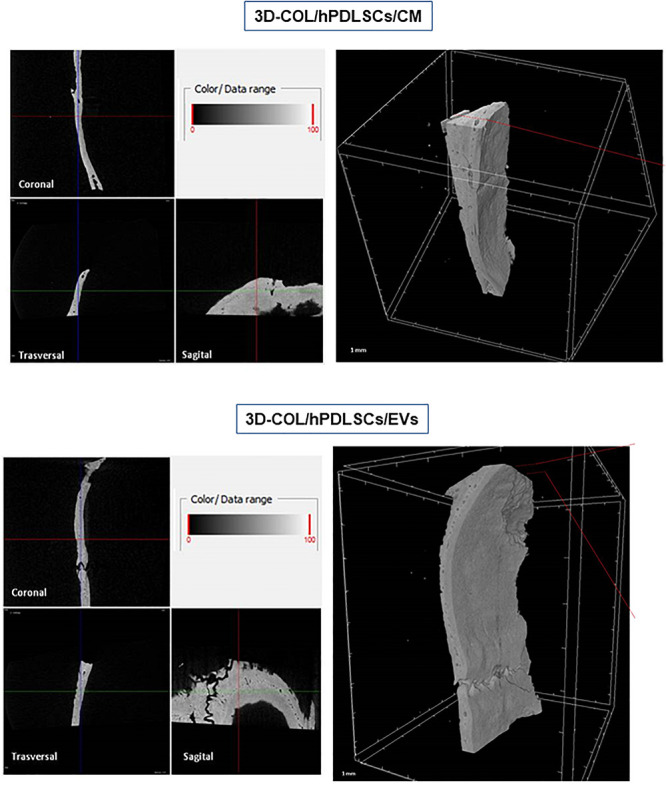
Micro-computed tomography (CT) analyses. 3D virtual histologic evaluation in volume rendering visualization modality of 3D-COL/hPDLSCs/CM and 3D-COL/hPDLSCs/EVs, with 2D virtual sectioning in the 3 orthogonal planes.

**Figure 8:**
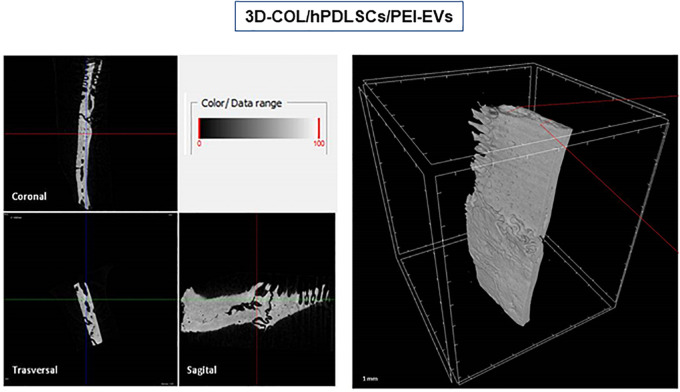
Micro-computed tomography (CT) analyses. 3D virtual histologic evaluation in volume rendering visualization modality of 3D-COL/hPDLSCs/PEI-EVs, with 2D virtual sectioning in the 3 orthogonal planes.

**Figure 9:**
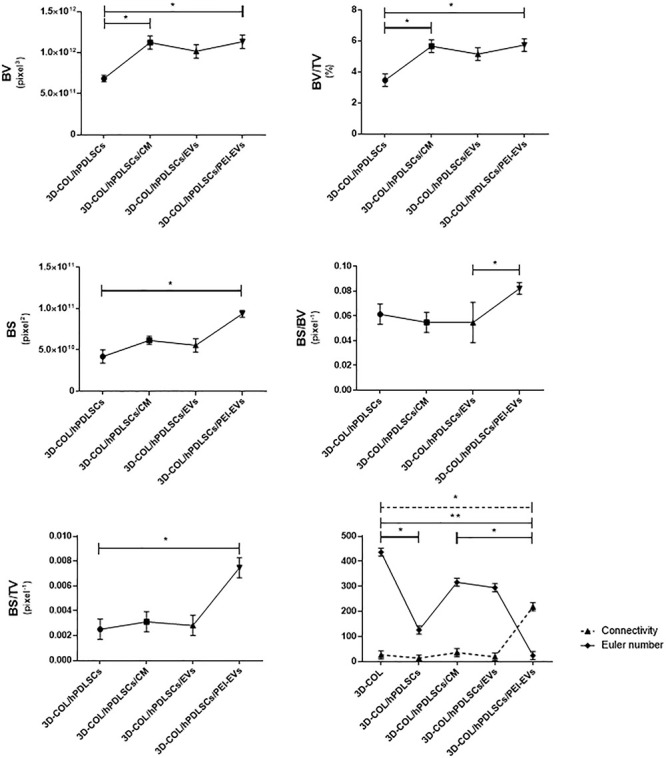
Morphometric analysis. BV, BV/TV, BS (2D), BS/BV, BS/TV, connectivity and Euler number evidenced a better regenerative process in 3D-COL/hPDLSCs/PEI-EVs group. ^∗^*P* < 0.05, ^∗∗^*p* < 0.01.

*Ex vivo* MicroCT imaging showed the regeneration level in the different examined groups (Figure 6–8). Two and three-dimensional sections revealed the quantification of the membrane integration rate in 3D-COL and 3D-COL/hPDLSCs (Figure 6), 3D-COL/hPDLSCs/CM and 3D-COL/hPDLSCs/EVs (Figure 7), 3D-COL/hPDLSCs/PEI-EVs (Figure 8). In particular, 3D-COL/hPDLSCs/PEI-EVs showed an important regenerative process and integration level, evident in sagital cutting view. The quantification of bone parameters provided support for the results of the μCT images (Figure 9). The BV, BV/TV, BS, and BS/TV were significantly higher in the 3D-COL/hPDLSCs/PEI-EVs group compared to the group 3D-COL/hPDLSCs. The BS/BV was significantly higher in the 3D-COL/hPDLSCs/PEI-EVs group compared to 3D-COL/hPDLSCs/EVs. The connectivity was also significantly higher in the 3D-COL/hPDLSCs/PEI-EVs group compared to 3D-COL. On the contrary, the Euler number was significantly lower in the 3D-COL/hPDLSCs/PEI-EVs group compared to 3D-COL and 3D-COL/hPDLSCs/CM groups. However, a partial regeneration was shown also in the group 3D-COL/hPDLSCs/CM, given that BV and percent bone volume were increased compared to 3D-COL/hPDLSCs.

## Discussion

The regeneration of bone tissue is an aspect of primary importance in the medical field and the research aimed to develop new approaches in order to permit it. In particular, the use of scaffolds and biomaterials is arising as a promising strategy and important results have been obtained already. Moreover, the addiction of MSCs and their derivatives seems to enhance their beneficial effects (Diomede et al., 2016b; Gugliandolo et al., 2018). MSCs derived from oral cavity showed the differentiation ability and immunomodulatory properties (Diomede et al., 2018e; Pizzicannella et al., 2018b).

In previous works our group have already demonstrated the bone regenerative capacity of different scaffolds enriched with CM or EVs (Diomede et al., 2018c,d). In this work we evidenced the increased integration and greater bone regeneration capacity of 3D-COL/hPDLSCs/PEI-EVs compared to 3D-COL/hPDLSCs, 3D-COL/hPDLSCs/CM and 3D-COL/hPDLSCs/EVs. These results confirmed previous ones where we demonstrated that scaffolds enriched with hPDLSCs and PEI-EVs showed better osteogenic properties both *in vitro* and *in vivo* compared to EVs. In particular, PEI-EVs activated an osteogenic response with the up-regulation of osteogenic genes and the increased protein levels of BMP2/4 (Diomede et al., 2018a). In this work we confirmed the major osteogenic ability of the enrichment with PEI-EVs compared not only with EVs but also compared with CM.

Polyethylenimine is a cationic polymer that we used to coat EVs and it is well studied as delivery system for nucleic acids (Pandey and Sawant, 2016). In a previous work we have already demonstrated that PEI-EVs were internalized at cytoplasmic level with higher efficiency compared to EVs alone and were more efficacious in inducing differentiation toward the osteogenic lineage and bone regeneration (Diomede et al., 2018a). In fact, a significant increase of osteogenic genes, such as TGFB1, MMP8, TUFT1, TFIP11, BMP2, and BMP4, in the presence of PEI-EVs was evidenced. The upregulation of BMP2/4 was confirmed for collagen membrane enriched with PEI-EVs and hPDLSCs both *in vitro* by Western blot and *in vivo* by immunofluorescence (Diomede et al., 2018a).

Also in this work PEI-EVs showed a better performance. Indeed, PEI-EVs showed a higher concentration at cytoplasmic level in hPDLSCs compared with non-engineered EVs. Likely this effect is connected with PEI capability to promote, by its proton-sponge effect, cellular uptake without the need for an additional endosomolytic agent (Zuber et al., 2001).

We observed a significant increase of gene expression of RUNX-2, BMP2/4 and COL1A1 in hPDLSCS cultured in the presence of the 3D-COL and PEI-EVs *in vitro* indicating an improved osteogenic process. In association, VEGF and VEGFR2 gene expression increased *in vitro* and the protein levels of VEGF and VEGFR2 were higher in 3D-COL/hPDLSCs/PEI-EVs compared to the other groups both *in vitro* and *in vivo*.

Interestingly, it was reported that RUNX-2 is a component of the genetic program that modulate the expression of VEGF during endochondral bone formation (Zelzer et al., 2001). Furthermore, BMP-2 was found to be able to promote *in vitro* angiogenesis in human endothelial progenitor cells (Chen et al., 2018) and BMP-2 treatment enhanced VEGFA expression in adipose stem cells grown on biphasic calcium phosphate (Overman et al., 2013). In addition, BMP-4 increased the secretion of VEGF in adult retinal pigment epithelium-19 and in osteoblast-like MC3T3-E1 cells (Kozawa et al., 2001; Vogt et al., 2006). These data may indicate that the increase of RUNX-2 and BMP2/4 may drive the increase of VEGF and of its receptor. Moreover, it has been shown that VEGF can act in a synergistic way together with BMP4 in order to increase cartilage formation in the early stages of endochondral bone formation, leading to a significant enhancement of bone formation and bone healing. However, this effect depended on the ratio between VEGF and BMP4, indeed an improper ratio caused detrimental effects on bone healing. Then, VEGF plays an important role in bone formation elicited by BMP4, and it can significantly enhance BMP4-elicited bone formation and regeneration through multiple mechanisms (Peng et al., 2002). Zhang et al. found that sustained BMP signaling in osteoblasts increased VEGFA production and increase bone and vessels (Zhang et al., 2009).

Then, our results evidenced the capacity of 3D-COL/hPDLSCs/PEI-EVs to increase the levels of the pro-angiogenic factor VEGF that was shown to play an important role in osteogenesis and bone regeneration. The positive role of VEGF on osteogenesis has been demonstrated already (Casap et al., 2008; Wang et al., 2011; Xu et al., 2018) and VEGF may regulate also calvarial ossification (Zelzer et al., 2002). Vascularization is a fundamental process during osteogenesis and bone regeneration. Suggesting the important role of VEGF, its expression was evidenced during bone repair (Ferguson et al., 1999). Moreover, it was reported that VEGF administration in an experimental model of osteonecrosis of the femoral head led to bone remodeling and new bone formation (Dailiana et al., 2018). The loss of VEGF in Osterix positive osteoblast progenitor cells caused a reduction of calvaria ossification, indicating that VEGF derived from these cells is required for optimal intramembranous bone formation (Duan et al., 2016). The reduction of VEGF injured endochondral bone formation, decreasing angiogenesis and osteogenesis, causing a delay in fracture healing (Ding et al., 2018). A work evidenced also that the administration of MSCs and VEGF stimulation on days 1–14 and 1–21 showed more bone formation compared with the group receiving only MSCs (Dreyer et al., 2017). VEGF-transfected adipose-derived stromal cells implanted with bone marrow stromal cells and hydroxyapatite/β-tricalcium phosphate granules in rats with critical size calvarial defects showed better bone regeneration and vascularization that bone marrow stromal cells alone (Kang et al., 2017). However, our construct was able to increase the levels not only of VEGF, but also of its receptor VEGFR2. Interestingly, the effects induced by VEGF on osteoblast differentiation seem to be mediated by VEGFR2 (Duan et al., 2015). Then, we can suggest that the increase of RUNX-2 and BMP2/4 promoted by PEI-EVs may induce the increase of VEGF, that in turn, through the activation of VEGFR2 may exert a positive role on osteogenesis.

Our group has evidenced the upregulation of miR-210 and VEGF in hPDLSCs cultured in the presence of Endobon^®^ Xenograft Granules (G), a fully deproteinated hydroxyapatite ceramic scaffold derived from cancellous bovine bone. Also VEGF release increased in the cells cultured with the biomaterial (Pizzicannella et al., 2018a).

In accordance with the increase of VEGF, the histological analysis evidenced the presence of vascularization processes in the group 3D-COL/hPDLSCs/PEI-EVs. This group showed the better performance in terms of bone regenerative capacity. This result was confirmed also by microCT analysis. The bone morphometric analysis with microCT evidenced the better bone regeneration in 3D-COL/hPDLSCs/PEI-EVs. In particular, the results indicated a higher BV and BS, and confirmed also by the increase of BS/TV, BS/BV, BV/TV. For both bone quantity and quality we evidenced an improvement in the group that received 3D-COL/hPDLSCs/PEI-EVs, given the higher percentage of bone and the higher connectivity.

The new vessels, other than provide nutrients and oxygen necessary for osteogenesis, may produce osteogenic factors by endothelial cells (Grosso et al., 2017).

In synthesis the 3D-COL/hPDLSCs/PEI-EVs experimental group increased the expression of proangiogenic molecules as VEGF and VEGFR2 that could exert beneficial regenerative effects contributing directly to osteogenesis, activating a local angiogenic response through autocrine/paracrine effects. The same machinery occurs during embryonic development when the VEGFA released from mesenchyme promote together bFGF and angiopoietin the sprouting process.

Sprouting angiogenesis represents a dynamic process in which endothelial cells in hierarchically manner migrate and form new tubes and new connections, through the develop of a functionally vascular network. During development endothelial cells assume two distinct cellular phenotypes: tip and stalk cells with specialized functions and different gene expression patterns and VEGF signaling drives tip cell migration and stalk cell proliferation (Blanco and Gerhardt, 2013). These data evidenced the key role of the VEGF and its receptor and indicate an innovative biocompatible system potentially useful in the reconstructing of calvaria defects in tissue engineering.

## Conclusion

In conclusion our results evidenced that 3D-COL/hPDLSCs/PEI-EVs may be an efficacious strategy to induce bone regeneration and vascularization of bone defects, thanks to its capacity to increase the levels of VEGF.

## Ethics Statement

The study was performed in accordance with the guidelines of the Helsinki Declaration (2013). The written approval for the human periodontal ligament collection performed in this study has been obtained from the Medical Ethics Committee at the Medical School, “G. d’Annunzio” University of Chieti–Pescara, Chieti, Italy (n°266/17.04.14, Principal investigator: OT). The written informed consent, for clinical research and for the processing of personal data, was obtained from all subjects before sample collection. The Department of Medical, Oral and Biotechnological Sciences and the Laboratory of Stem Cells and Regenerative Medicine are certified according to the quality standard ISO 9001:2008 RINA (certificate no. 32031/15/S). Ethics statement for animal use. All animal care and use was accomplished according to the European Organization Guidelines for Animal Welfare. The study has been authorized by the Ministry of Health “General Direction of animal health and veterinary drug” (Authorization 768/2016-PR 28/07/2016- D.lgs 26/2014). The experiments were planned in such a way to minimize the total number of rats needed for the study.

## Author Contributions

AG, FD, and JP carried out the experiments, analyzed the results, and wrote the manuscript. TO performed the micro-CT. AV performed the vesicles engineering and evaluation. PB, AF, EM, and OT designed the study and revised the manuscript.

## Conflict of Interest Statement

The authors declare that the research was conducted in the absence of any commercial or financial relationships that could be construed as a potential conflict of interest.

## Footnotes

1
www.doubling-time.com/

## Supplementary Material

FIGURE S1 Entire original western blot.

FIGURE S2 Power analysis using G^∗^Power software.

FIGURE S3 Confocal laser scanning microscopy figure.
