# Addendum: Engineered extracellular vesicles from human periodontal-ligament stem cells increase VEGF/VEGFR2 expression during bone regeneration

**DOI:** 10.3389/fphys.2023.1148929

**Published:** 2023-03-23

**Authors:** Jacopo Pizzicannella, Agnese Gugliandolo, Tiziana Orsini, Antonella Fontana, Alessia Ventrella, Emanuela Mazzon, Placido Bramanti, Francesca Diomede, Oriana Trubiani

**Affiliations:** ^1^ Department of Medical, Oral and Biotechnological Sciences, “G. d'Annunzio” University of Chieti–Pescara, Chieti, Italy; ^2^ IRCCS Centro Neurolesi Bonino Pulejo, Messina, Italy; ^3^ Institute of Cell Biology and Neurobiology, National Research Council, Rome, Italy; ^4^ Department of Pharmacy, “G. d'Annunzio” University of Chieti–Pescara, Chieti, Italy

**Keywords:** mesenchymal stem cells, bone regeneration, VEGF, VEGFR2, collagen membrane, extracellular vesicles, polyethylenimine

In the published article, we would to like to add the merge relative to panel C1 and C2 of the Figure 2 as Supplementary Figure S3.

The authors apologize for this error and state that this does not change the scientific conclusions of the article in any way. The original article has been updated.

## Supplementary material

Supplementary Figure S3 Confocal laser scanning microscopy figure.

