# Supplementary figures and images for: Engineered Extracellular Vesicles From Human Periodontal-Ligament Stem Cells Increase VEGF/VEGFR2 Expression During Bone Regeneration
Source: Front Physiol. 2019 Apr 30;10:512. doi: 10.3389/fphys.2019.00512 (PMC6503111; doi:10.3389/fphys.2019.00512)

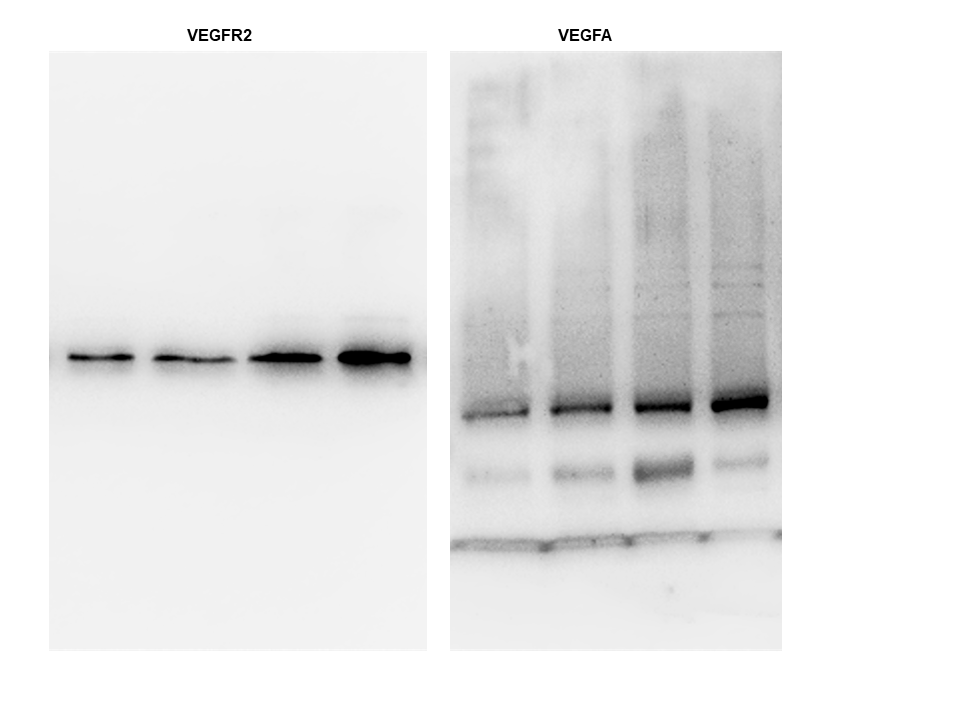

Supplement: FIGURE S1 — Entire original western blot. [file Image_1.TIF]

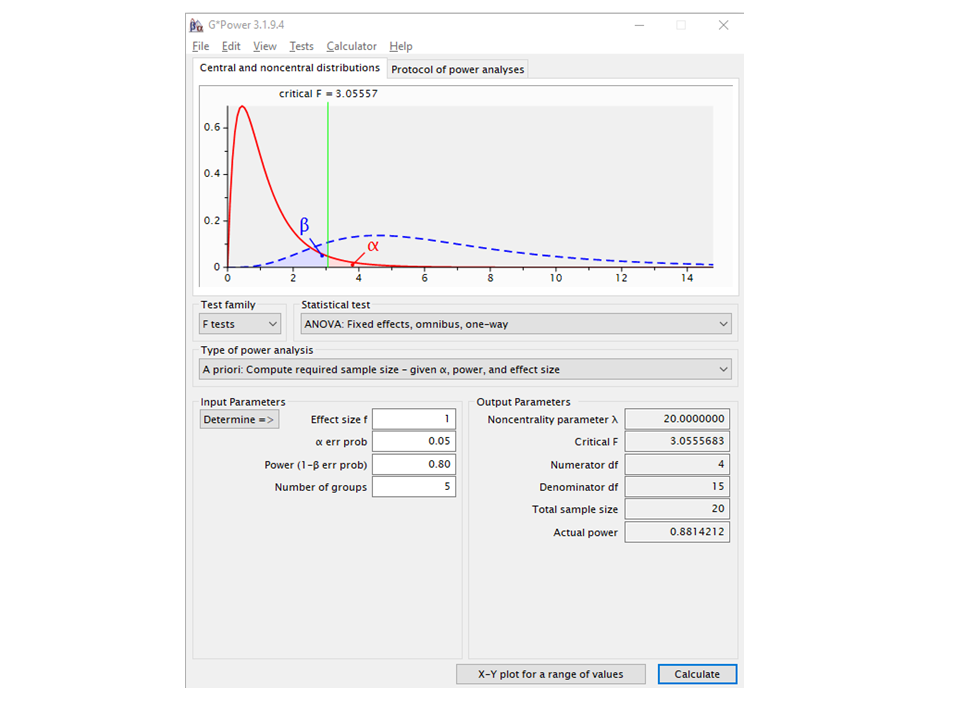

Supplement: FIGURE S2 — Power analysis using G∗Power software. [file Image_2.TIF]

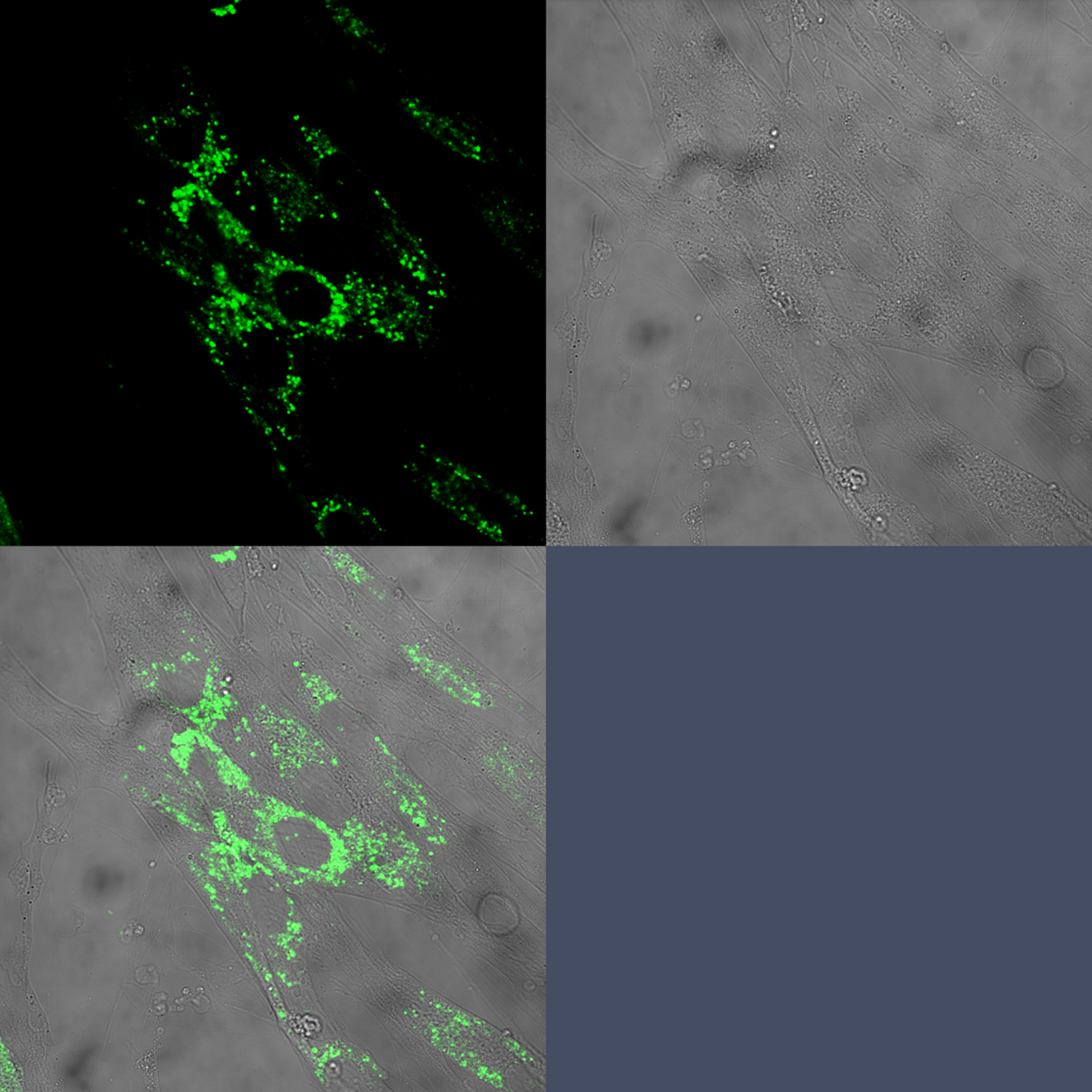

Supplement: FIGURE S3 — Confocal laser scanning microscopy figure. [file Image_3.tif]
