# Supplementary figures and images for: Addendum: Engineered extracellular vesicles from human periodontal-ligament stem cells increase VEGF/VEGFR2 expression during bone regeneration
Source: Front Physiol. 2023 Mar 23;14:1148929. doi: 10.3389/fphys.2023.1148929 (PMC10078805; doi:10.3389/fphys.2023.1148929)

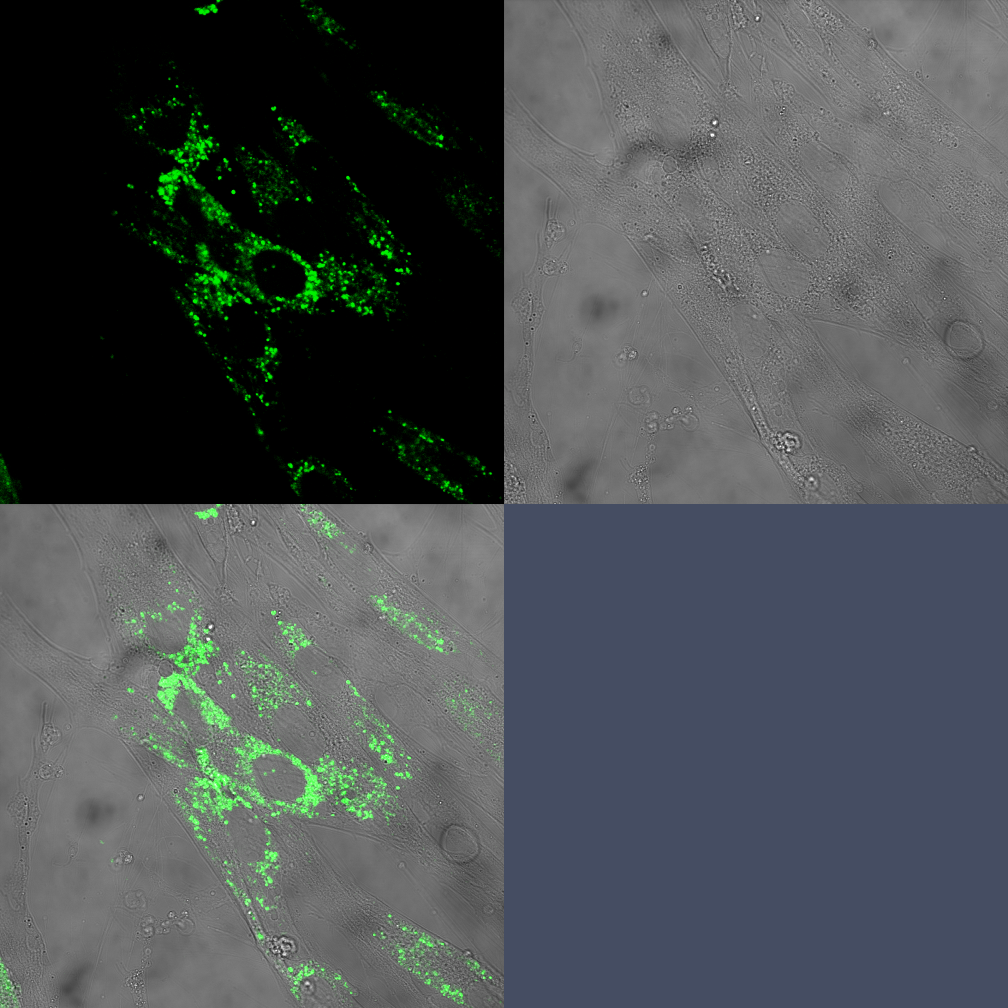

Supplement: Supplementary file 1 [file Image3.tif]
